# Treatment with trastuzumab deruxtecan in patients with HER2-positive breast cancer and brain metastases and/or leptomeningeal disease (ROSET-BM)

**DOI:** 10.1038/s41523-023-00584-5

**Published:** 2023-10-11

**Authors:** Naoki Niikura, Takashi Yamanaka, Hironori Nomura, Kazuhiro Shiraishi, Hiroki Kusama, Mitsugu Yamamoto, Kazuo Matsuura, Kenichi Inoue, Sachiko Takahara, Shosuke Kita, Miki Yamaguchi, Tomoyuki Aruga, Nobuhiro Shibata, Akihiko Shimomura, Yuri Ozaki, Shuji Sakai, Yoko Kiga, Tadahiro Izutani, Kazuhito Shiosakai, Junji Tsurutani

**Affiliations:** 1 Department of Breast Oncology, Tokai University School of Medicine, Kanagawa, Japan; 2 Department of Breast and Endocrine Surgery, Kanagawa Cancer Center, Kanagawa, Japan; 3 First Department of Surgery, University of the Ryukyus, School of Medicine, Okinawa, Japan; 4 Department of Medical Oncology, Nagoya Medical Center, Nagoya, Aichi Japan; 5 Department of Breast and Endocrine Surgery, Osaka International Cancer Institute, Osaka, Japan; 6 Department of Breast Surgery, Hokkaido Cancer Center, Hokkaido, Japan; 7 Department of Breast Oncology, Saitama Medical University International Medical Center, Saitama, Japan; 8 Division of Breast Oncology, Saitama Cancer Center, Saitama, Japan; 9 Department of Breast Surgery, Kitano Hospital, Osaka, Japan; 10 Department of Medical Oncology, National Cancer Center Hospital, Tokyo, Japan; 11 Department of Breast Surgery, JCHO Kurume General Hospital, Fukuoka, Japan; 12 Department of Breast Surgery, Komagome Hospital, Tokyo, Japan; 13 Cancer Treatment Center, Kansai Medical University Hospital, Osaka, Japan; 14 Department of Breast and Medical Oncology, National Center for Global Health and Medicine, Tokyo, Japan; 15 Department of Breast Oncology, Aichi Cancer Center Hospital, Nagoya, Aichi Japan; 16 Department of Imaging Diagnosis and Nuclear Medicine, Tokyo Women’s Medical University, Tokyo, Japan; 17 Oncology Medical Science Department, Daiichi Sankyo Co., Ltd., Tokyo, Japan; 18 Data Intelligence Department, Daiichi Sankyo Co., Ltd., Tokyo, Japan; 19 Advanced Cancer Translational Research Institute, Showa University, Tokyo, Japan

**Keywords:** Breast cancer, Metastasis, Metastasis, Outcomes research

## Abstract

Therapeutic options for breast cancer patients with brain metastases (BM)/leptomeningeal carcinomatosis (LMC) are limited. Here, we report on the effectiveness and safety of trastuzumab deruxtecan (T-DXd) in human epidermal growth factor receptor 2-positive breast cancer patients with BM. Data were analyzed for 104 patients administered T-DXd. Overall response rate (ORR), progression-free survival (PFS), overall survival (OS), intracranial (IC)-ORR, and IC-PFS were evaluated. ORR by investigator assessment was 55.7% (total population). Median PFS was 16.1 months; 12-month OS rate was 74.9% (total population). Median time-to-treatment failure was 9.7 months. In 51 patients with BM imaging, IC-ORR and median IC-PFS by independent central review were 62.7% and 16.1 months, respectively. In 19 LMC patients, 12-month PFS and OS rates were 60.7% and 87.1%, respectively. T-DXd showed effectiveness regarding IC-ORR, IC-PFS, PFS, and OS in breast cancer patients with BM/active BM, and sustained systemic and central nervous system disease control in LMC patients.

*Trial Registration*: UMIN000044995.

## Introduction

Breast cancer is one of the most common causes of central nervous system (CNS) metastases, including brain metastases (BM) and leptomeningeal carcinomatosis (LMC), and patients with CNS metastasis have a poor prognosis. The molecular subtype of breast cancer has been shown to have an impact on the incidence of CNS metastases. For example, the incidence of CNS metastasis was found to be twice as high in patients with human epidermal growth factor receptor 2 (HER2)-positive breast cancer compared with HER2-negative breast cancer (21.8% vs 11.1%, respectively)^1^. For BM specifically, the incidence has been reported to be as high as 31% in patients with HER2-positive breast cancer^2^. Breast cancer patients with BM and/or LMC have limited treatment options.

Guidelines for breast cancer treatment recommend local therapy for patients with symptomatic BM, such as surgical resection or irradiation, and to continue with the same anti-HER2 therapy if the systemic clinical benefit persists^3–6^. The European Association of Neuro-Oncology–European Society for Medical Oncology guidelines recommend systemic therapy based on the primary tumor for most breast cancer patients, but there is a lack of evidence to support this^7^.

Based on the results of the HER2CLIMB trial (NCT02614794), combination therapy with tucatinib plus trastuzumab plus capecitabine was approved by the Food and Drug Administration (FDA) for the treatment of adult patients with unresectable, advanced, or metastatic HER2-positive breast cancer, including patients with BM, who have received at least one regimen of anti-HER2 therapy for metastatic breast cancer^8,9^. In the DESTINY-Breast01 study (NCT03248492), trastuzumab deruxtecan (T-DXd) showed clinical activity in breast cancer patients with BM who had received ≥2 prior anti-HER2-based regimens or who were previously treated with trastuzumab emtansine (T-DM1)^10,11^. The DESTINY-Breast03 trial showed higher progression-free survival (PFS) and overall response rate (ORR) in HER2-positive breast cancer patients with BM who were treated with T-DXd compared with those treated with T-DM1^12,13^. The results of the TUXEDO-1 and DEBBRAH trials also support the use of T-DXd in HER2-positive breast cancer patients with BM^14,15^.

Of note, only patients with stable BM were included in the DESTINY-Breast01 and 03 studies (*n* = 24 and *n* = 43, respectively), and sample sizes of patients with active BM in the TUXEDO-1 and DEBBRAH trials (*n* = 15 and *n* = 13, respectively) were small compared with the HER2CLIMB trial (174 patients with active BM). There is currently a lack of solid evidence on the activity of T-DXd in CNS metastases, and real-world data for T-DXd would be particularly valuable due to weak prospective evidence.

This study aimed to evaluate the effectiveness and safety of T-DXd for HER2-positive breast cancer patients with BM, including those with symptomatic and asymptomatic BM and those with active and stable BM or LMC, in the real-world clinical setting.

## Results

### Patients

**Fig. 1:**
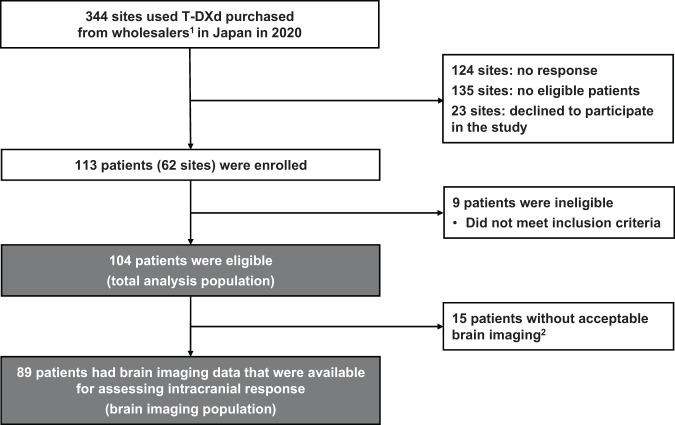
Patient disposition. ^1^This is the result of a survey by Daiichi Sankyo Co., Ltd. of ENHERTU^®^ purchases at medical institutions and is not information from medical institutions. ^2^No brain imaging data were submitted: 1 patient. No brain imaging data after initiation of T-DXd were available: 14 patients. *BC* breast cancer, *BM* brain metastasis, *T-DXd* trastuzumab deruxtecan.

Among 344 sites that were surveyed for participation in the present study, 220 sites responded. A total of 113 HER2-positive breast cancer patients with BM (from 62 sites) who were treated with T-DXd were enrolled in this study (Fig. 1). Among them, 104 patients were included in the total population and 89 patients were included in the population with imaging data of the brain lesion. The median follow-up duration was 11.2 (range, 0.9–17.0) months.

The background patient characteristics are summarized in Table 1. Symptomatic BM was observed in 32/104 (30.8%) patients. Among the total population (*N* = 104*)*, 73 (70.2%) patients had active BM without LMC, 17 (16.3%) had active BM with LMC, 6 (5.8%) had stable BM, 2 (1.9%) had only LMC, and 6 (5.8%) were not classified (image not evaluated). In total, 41 (39.4%) patients were still under treatment.

**Table 1:** Baseline characteristics (total population).

Characteristic	Value
	*N* = 104
*Sex*	
Male/female	1 (1.0)/103 (99.0)
*Age, years*	
<65/ ≥ 65	75 (72.1)/29 (27.9)
*HER2 status (IHC)*^*a*^	
0, 1 + /2 + /3+	0 (0.0)/18 (17.3)/84 (80.8)
Unknown	2 (1.9)
*HER2 status (ISH)*	
Positive/negative	29 (27.9)/1 (1.0)
Unknown	74 (71.2)
*Estrogen receptor status*	
Positive/negative	59 (56.7)/44 (42.3)
Unknown	1 (1.0)
*Progesterone receptor status*	
Positive/negative	43 (41.3)/61 (58.7)
Unknown	0 (0.0)
Surgery for primary breast cancer	71 (68.3)
*Number of prior therapies for MBC*	
0–2CT	25 (24.0)
≥3	79 (76.0)
Median (Q1, Q3)	4.0 (3.0, 7.0)
*Prior treatment for MBC*	
Trastuzumab	94 (90.4)
Pertuzumab	88 (84.6)
Trastuzumab emtansine	91 (87.5)
Lapatinib	37 (35.6)
Time from initial CNS diagnosis to start of T-DXd, months, mean ± SD	22.6 ± 22.4
*ECOG PS*	
0/1/2/3–4	27 (26.0)/54 (51.9)/12 (11.5)/4 (3.8)
Unknown	7 (6.7)
Visceral metastasis except the brain	79 (76.0)
*Clinical presentation of BM*	
Symptomatic	32 (30.8)
Asymptomatic	72 (69.2)
*Drug used for symptoms of BM*	
Steroids	15 (14.4)
Anti-epileptics	11 (10.6)
*Local treatment for BM*^*b*^	
Treated	99 (95.2)
Whole-brain radiation	56 (53.8)
Within 30 days	6 (5.8)
Stereotactic irradiation	64 (61.5)
Surgery to remove a tumor	27 (26.0)
Untreated	5 (4.8)
*Classification of BM by ICR*	
Active BM	90 (86.5)
Without LMC	73 (70.2)
With LMC	17 (16.3)
Stable BM	6 (5.8)
Only LMC	2 (1.9)
Image not classified	6 (5.8)
*Number of BM*	
1	18 (17.3)
2–4	28 (26.9)
5–9	17 (16.3)
≥10	27 (26.0)
*Size of BM, cm (n* *=* *55)*	
Mean ± SD	2.1 ± 0.9
*Karnofsky PS*	
0–40	3 (2.9)
50–70	22 (21.2)
80–100	45 (43.3)
Unknown	34 (32.7)
GPA score	
0–1	0 (0.0)
1.5–2.0	9 (8.7)
2.5–3.0	43 (41.3)
3.5–4.0	18 (17.3)
Unknown	34 (32.7)

Data are no. (%), unless otherwise stated.

*BM* brain metastasis, *CNS* central nervous system, *ECOG* Eastern Cooperative Oncology Group, *GPA* Graded Prognostic Assessment, *HER2* human epidermal growth factor receptor 2, *ICR* Independent Central Review, *IHC* immunohistochemistry, *ISH* in situ hybridization, *LMC* leptomeningeal carcinomatosis, *MBC* metastatic breast cancer, *PS* performance status, *SD* standard deviation, *T-DXd* trastuzumab deruxtecan.

^a^HER2 status was based on the primary tumor. Two patients with IHC unknown were ISH+. One patient was IHC2+ and ISH−, but the brain lesion removed by surgery was IHC3+.

^b^Includes patients who have received multiple local treatments.

### Outcomes

**Fig. 2:**
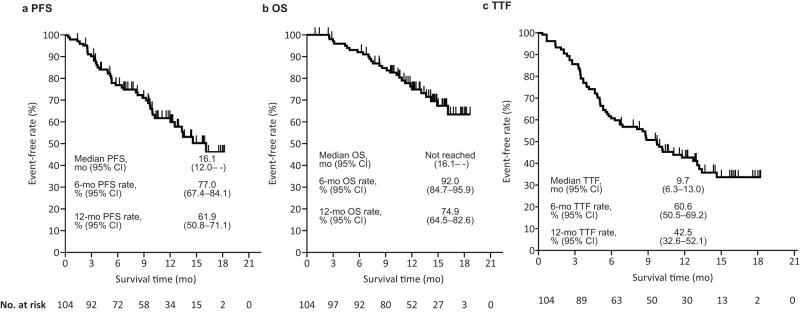
PFS, OS, and TTF (total population). **a** PFS, (**b**) OS, (**c**) TTF. *CI* confidence interval, *mo* month, *OS* overall survival, *PFS* progression-free survival, *TTF* time-to-treatment failure.

In the total population (*N* = 104*)*, the median PFS was 16.1 months (95% confidence interval [CI], 12.0%– -) (Fig. 2A), and the 12-month overall survival (OS) rate was 74.9% (95% CI, 64.5–82.6%) (Fig. 2B). The OS was immature because of the short observation period, and an additional analysis at 12 months is currently underway.

The median time-to-treatment failure (TTF) was 9.7 months (95% CI, 6.3–13.0%) (Fig. 2C). Discontinuation of treatment at the time of data cut-off was observed in 57/104 (54.8%) patients, with progressive disease causing the most discontinuations (26 [25.0%]) (Supplementary Table 1). Treatment was also discontinued due to adverse events (AEs) in 23 (22.1%) patients (10 [9.6%] patients had Grade ≥3 AEs), among whom 19 (18.3%) presented with interstitial lung disease (ILD) or lung disorders. There were 5 (4.8%) and 2 (1.9%) patients who presented with Grade 3, and Grade 4 ILD or lung disorders, respectively.

The ORR based on investigator assessment was 55.7% (complete response [CR]: 5.2%) in the total population (*n* = 97), 51.7% (CR: 6.9%) in symptomatic BM patients (*n* = 29), and 57.4% (CR: 4.4%) in asymptomatic BM patients (*n* = 68) (Supplementary Table 2).

**Fig. 3:**
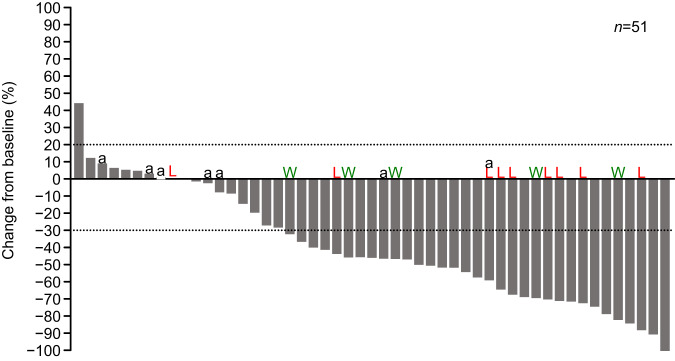
Waterfall plot of change in tumor size of the brain lesion (brain imaging population). ^a^Human epidermal growth factor receptor 2 immunohistochemistry is not 3+, unknown, or missing. *L* leptomeningeal carcinomatosis, *W* whole brain radiotherapy within 30 days.

A total of 51 patients with brain lesion imaging data were included in the intracranial (IC) evaluation by independent central review (ICR). The best overall response based on ICR assessment is summarized in Table 2. The median (95% CI) duration (days) from the start of T-DXd treatment to the first brain imaging was 56.0 (43.0–64.0) days. In the population with imaging data of the brain lesions (*n* = 51), the IC-ORR was 62.7% (Table 2). The median IC-duration of response (DOR) was not reached, the 12-month IC-DOR rate was 74.0%, and the median IC-PFS was 16.1 months (12.2– -). The IC-clinical benefit rate (CBR) was 70.6% at 6 months. A waterfall plot of the change in tumor size of brain lesion is shown in Fig. 3.

**Table 2:** IC-ORR based on independent central review (brain imaging population).

	IC-CR No. (%)	IC-PR No. (%)	IC-SD No. (%)	IC-PD No. (%)	IC-ORR % (95% CI)	IC-CBR at 6 months % (95% CI)
Total (*n* = 51)^a^	0 (0.0)	32 (62.7)	16 (31.4)	3 (5.9)	62.7 (48.1–75.9)	70.6 (56.2–82.5)
*Analytical classification of BM*						
Analytical active BM (*n* = 37)	0 (0.0)	20 (54.1)	16 (43.2)	1 (2.7)	54.1 (36.9–70.5)	62.2 (44.8–77.5)
Analytical stable BM (*n* = 5)	0 (0.0)	5 (100.0)	0 (0.0)	0 (0.0)	100.0 (47.8–100.0)	100.0 (47.8–100.0)
LMC (*n* = 9)	0 (0.0)	7 (77.8)	0 (0.0)	2 (22.2)	77.8 (40.0–97.2)	88.9 (51.8–99.7)
*Clinical presentation of BM*						
Symptomatic (*n* = 19)	0 (0.0)	11 (57.9)	7 (36.8)	1 (5.3)	57.9 (33.5–79.7)	63.2 (38.4–83.7)
Asymptomatic (*n* = 32)	0 (0.0)	21 (65.6)	9 (28.1)	2 (6.3)	65.6 (46.8–81.4)	75.0 (56.6–88.5)
*Steroid use at baseline*						
Yes (*n* = 11)	0 (0.0)	5 (45.5)	5 (45.5)	1 (9.1)	45.5 (16.7–76.6)	54.5 (23.4–83.3)
No (*n* = 40)	0 (0.0)	27 (67.5)	11 (27.5)	2 (5.0)	67.5 (50.9–81.4)	75.0 (58.8–87.3)

*BM* brain metastasis, *CBR* clinical benefit rate, *CI* confidence interval, *CR* complete response, *IC* intracranial, *LMC* leptomeningeal carcinomatosis, *ORR* overall response rate, *PD* progressive disease, *PR* partial response, *SD* stable disease.

^a^Of the 89 patients, 12 patients had no target lesions and 26 patients were not evaluable for intracranial response.

The incidence of time-to-deterioration of CNS metastasis-related symptoms was 85.2% (95% CI, 74.5–91.7%) at 12 months, and the median time-to-deterioration of CNS metastasis-related symptoms was not reached (- – -) in the total population (*N* = 104) (Supplementary Fig. 1).

### Subgroup analysis

**Fig. 4:**
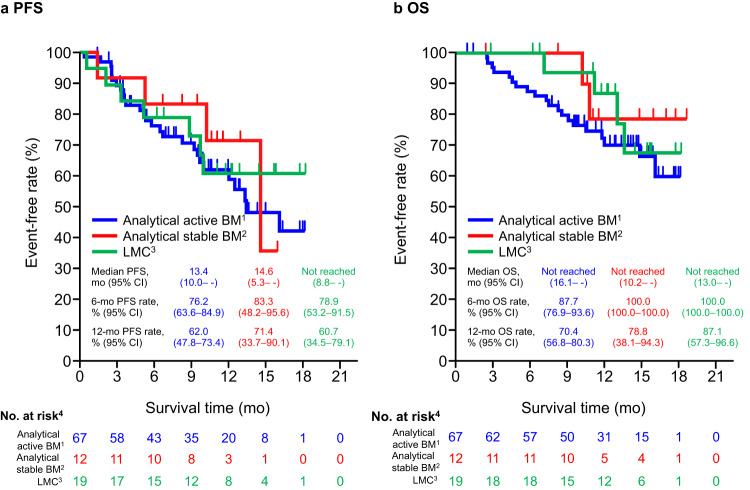
PFS and OS by classification of BM (total population). **a** PFS, (**b**) OS. ^1^Active (not including whole brain radiotherapy within 30 days). ^2^Stable + Active with whole brain radiotherapy within 30 days. ^3^Active with LMC/LMC only. ^4^Of the 104 patients, 6 patients with brain imaging data at baseline were not classified by Independent Central Review. *BM* brain metastasis, *CI* confidence interval, *LMC* leptomeningeal carcinomatosis, *mo* months, *OS* overall survival, *PFS* progression-free survival.

The PFS and OS by breast cancer-specific Graded Prognostic Assessment (GPA) score^16,17^ are shown in Supplementary Fig. 2. Patients with poor prognosis by GPA did not differ from those with good prognosis by GPA in terms of PFS and OS. Furthermore, there were no differences in PFS and OS between patients with active BM, stable BM, and LMC. The PFS and OS by classification of BM are shown in Fig. 4A and B, respectively. The median IC-PFS was 16.1 months (95% CI, 11.8%– -) in patients with analytical active BM (*n* = 57). The IC-ORR (*n* = 51) was 62.7% (95% CI, 48.1–75.9%), and the median IC-PFS (*n* = 89) was 16.1 months (95% CI, 12.2%– -) in the patients with evaluable IC imaging (Supplementary Fig. 3).

### LMC

The current data set included 19 patients with LMC (17 patients with BM, 2 patients without BM), with 12-month PFS and OS rates of 60.7% (95% CI, 34.5–79.1%) and 87.1% (95% CI, 57.3–96.6%), respectively. Nine patients with imaging data of the brain lesion were included in the IC evaluation by ICR. In patients with BM and LMC, the IC-ORR was 77.8% (7/9 patients).

## Discussion

This was a study of 104 HER2-positive breast cancer patients with BM who were treated with T-DXd in a real-world clinical setting. Our findings suggest that T-DXd may be a treatment option for HER2-positive breast cancer patients with BM, including patients with LMC and active and stable BM, who are characterized by poor performance status.

In previous studies of the tyrosine kinase inhibitors lapatinib and neratinib in HER2-positive breast cancer patients with BM, PFS was less than 6 months and severe disease was not well controlled^18,19^. In the phase 2 LANDSCAPE trial, lapatinib and capecitabine for asymptomatic and oligosymptomatic patients with BM showed a response rate of 66% and time to whole brain radiotherapy of 8.3 months^20^. Tucatinib, trastuzumab, and capecitabine combination showed a response rate of 47.3% compared with 20% in the trastuzumab and capecitabine group among patients with active BM in the HER2CLlMB study^9^. Median PFS was improved from 4.1 months to 9.5 months, and OS from 11.6 months to 20.7 months with the addition of tucatinib. Based on this study, the combination of tucatinib, trastuzumab, and capecitabine is now regarded as the preferred systemic treatment option for active BM in patients with HER2-positive breast cancer^4,5,21^.

Despite assumptions that monoclonal antibodies do not cross the blood–brain barrier, a radioisotope form of trastuzumab, imaging [64Cu] DOTA-trastuzumab, has been shown to localize in the BM of patients with HER2-positive metastatic breast cancer. In the PATRICIA study, pertuzumab plus high-dose trastuzumab (6 mg/kg weekly) in patients with progressive BM showed clinical benefit. The CNS ORR was 11% with four partial responses (median DOR, 4.6 months). The CBR at 4 and 6 months were 68% and 51%, respectively^22^. Clinical data on the potential activity of antibody–drug conjugates in stable and active BM were reported. The response rate with single-agent T-DM1 was 49.3% in the KAMILLA study, including the subset of patients with measurable BM without previous local radiotherapy^23^. In the DESTINY-Breast03 trial, which included 43 patients with stable BM, the PFS was 15 months, and the response rate was 80%^12^. The TUXEDO-1 trial was a single-arm prospective study of patients with newly diagnosed or progressive active BM treated with T-DXd. The response rate was 73.3% (11/15), median PFS was 14 months, and median OS was not reached at a median follow-up of 12 months^14^. The median PFS in the present study (16.1 months) was comparable to that in the BM subgroup of the DESTINY-Breast03 study (15 months) and numerically longer than that in the BM subgroup of the HER2CLIMB trial (9.5 months). Although the proportion of patients with BM was small, the IC-ORR was 62.7% in patients with measurable BM. The IC-CBR was 70.6% in the present study, suggesting that clinical benefit could be obtained in most patients. The median IC-PFS was higher (16.1 months) compared with that reported in the HER2CLIMB trial (9.9 months)^8,9^. In a recent retrospective multicenter cohort study of 17 patients with mainly HER2-positive metastatic breast cancer with BM^24^, the CNS-ORR and the 12-month CNS-PFS were reported to be 73% (11/15) and 74.7%, respectively, which is slightly higher than the IC-ORR and 12-month IC-PFS in the present study (62.7% and 64.3%, respectively).

Of note, our study included patients with poor Karnofsky performance status scores who are usually excluded from prospective trials, such as those with GPA scores of 1.5–2.0^16,17^. Our study showed no differences in PFS and OS between GPA score subgroups, meaning that T-DXd showed consistent effectiveness in terms of PFS and ORR in breast cancer patients with poor prognosis. The effectiveness of T-DXd for the treatment of IC lesions of breast cancer patients with active BM was similar to that in patients with stable BM, and T-DXd may provide some clinical benefit in patients with both active and stable BM.

Our data suggest a longer PFS in patients with LMC compared with that reported in previous studies. An analysis of the Epidemiological Strategy and Medical Economics MBC database showed a median OS of 5.6 months in patients with HER2-positive LMC^25^. In a previous phase 2 single-arm study that evaluated the efficacy of the combination of tucatinib plus trastuzumab and capecitabine in patients with HER2-positive breast cancer and newly diagnosed LMC, the median time to CNS progression was 6.9 months, and the median OS time was 11.9 months^26^. In the present study, both the median PFS and median OS in patients with LMC were not reached. PFS and OS at 12 months were 60.7% and 87.1%, respectively, in patients with LMC. These findings suggest that T-DXd was effective in patients with LMC, who were not responsive to local therapy and in whom pharmacotherapy was not expected to be effective. A recent retrospective study of eight evaluable patients with HER2-positive metastatic breast cancer and LMC showed a durable response with T-DXd^27^; 50% (4/8) of patients achieved partial response, 50% (4/8) had stable disease, and the median OS was 10.4 months. The response was centrally reviewed using the EORTC/RANO revised LM scorecard^28^. Of note, the DEBBRAH trial is still ongoing and is recruiting a small cohort of patients with LMC, which will provide prospective data on this rare subgroup^15^.

In this study, the most common AE leading to treatment discontinuation was ILD (18.3%), and this incidence of ILD was slightly higher than that reported in the DESTINY-Breast01 study (13.6%)^10^. This difference in the incidence of ILD is consistent with the results of an integrated analysis of DESTINY-Breast01 and DS8201-A-J101 (unpublished data) in which the incidence of ILD in Japanese patients was higher than that in the overall population. This is also supported by a pooled analysis of nine phase 1 and 2 T-DXd monotherapy studies in which enrollment in Japan was a significant risk factor for ILD^29^. No novel AEs leading to treatment discontinuation were observed in breast cancer patients with BM who were treated with T-DXd.

The present study has some limitations. This was not a prospective study. Because the presence or absence of BM was determined by the investigator, the possibility of reporting bias cannot be ruled out as to whether all BM patients were reported. LMC was diagnosed on imaging by ICR, and the presence of tumor cells in spinal fluid was not confirmed. The frequency of imaging evaluation was not specified, so the ORR and PFS may have been overestimated. For greater objectivity, the event onset in time-to-deterioration of CNS metastasis-related symptoms was defined as the date of treatment initiation and was not reported by physicians. Therefore, the impact of pharmacotherapy for symptom management may not have been properly assessed. In addition, as the response in patients with LMC was based on RECIST-based assessment of parenchymal BM lesion, the results regarding drug effectiveness should be interpreted with caution. Furthermore, the present study was conducted only in Japanese patients, which limits the generalizability of current findings to other ethnicities.

In conclusion, T-DXd was effective in breast cancer patients with BM, regardless of the presence of active or stable BM or the presence of LMC, indicating that IC tumors may be controlled by T-DXd. T-DXd has a manageable AE profile and may have survival benefits in breast cancer patients with BM who have a poor prognosis (low Karnofsky performance status score). In a small number of breast cancer patients with LMC, sustained systemic disease control was achieved with T-DXd. The ongoing DESTINY-Breast12 study (NCT04739761) will confirm the efficacy of T-DXd for active BM in patients with HER2-positive breast cancer.

## Methods

### Study design

This was a multicenter, retrospective, medical chart review study. To avoid selection bias, a questionnaire was sent to all 344 medical institutions in Japan regarding usage of T-DXd (ENHERTU^®^, Daiichi Sankyo Co., Ltd., Tokyo, Japan and AstraZeneca, Cambridge, UK) for the treatment of HER2-positive breast cancer especially in patients with BM as of December 31, 2020, of which 62 medical institutions had eligible patients for this study and agreed to participate in the study. Data from HER2-positive breast cancer patients with BM who received T-DXd treatment between May 25, 2020, and April 30, 2021, were collected from each participating institution using a medical record retrieval system. The principal investigators checked the medical records for all extracted patients, and using the continuous survey method, all the research patients who met all the inclusion criteria and did not fulfill the exclusion criteria were selected. The data cut-off date for survival and other information was October 31, 2021; data entry began on November 12, 2021, and information from medical records was entered retrospectively.

The protocol was approved by the centralized authority (Ethics Review Committee at Tokeikai Kitamachi Clinic, reference number: DSY08309), as well as the individual ethics committees at each study center (see Supplementary Table 3). The study was conducted in accordance with the Declaration of Helsinki and adhered to local ethical guidelines. The present study was conducted using an opt-out approach. As this study was non-interventional and non-invasive, the need to obtain informed consent from patients was waived as per local ethical guidelines. This study was registered at UMIN-CTR Clinical Trials under the identifier number UMIN000044995 (date of trial registration: July 29, 2021).

### Patients

The inclusion criteria were patients aged ≥20 years at the beginning of T-DXd treatment, with pathologically documented HER2-positive breast cancer, and with BM (including stable BM after local treatment, BM before local treatment, and symptomatic BM). Patients who expressed a desire not to participate in the study prior to data fixation and those who had received T-DXd from participation in a clinical trial were excluded.

### Outcomes

The following outcomes were evaluated for the total population: TTF, PFS, ORR based on investigator assessment, OS, and time-to-deterioration of CNS metastasis-related symptoms. The following outcomes were evaluated for the population with imaging data of the brain lesion: IC-ORR, IC-PFS, IC-DOR, and IC-CBR. Each of the outcomes is defined in Supplementary Table 4.

### ICR and analytical classification

Brain imaging data (i.e., MRI or CT) of brain lesions from study patients were subjected to ICR to determine the effectiveness of treatment with T-DXd. The ICR consisted of three independent radiologists (a chairperson and two members) who were skilled in Response Evaluation Criteria in Solid Tumors (RECIST) version 1.1 evaluation to maintain a certain standard of evaluation. Two ICR members evaluated half of the images each, and the chairperson made an overall judgment of the results. All brain images are collected centrally. For each study patient, comprehensive assessment data at the time of visit and the date of imaging at each time point (visits at which response or progression was confirmed/not confirmed) were determined by ICR. BM was classified into analytical active BM, analytical stable BM, and LMC by the ICR as follows. The detailed criteria are shown in Supplementary Table 5.

### Analytical active BM by ICR

According to ICR, patients with increasing/growing tumors in two brain imaging comparisons before T-DXd administration were defined as “active by ICR”. In this study, those who had not undergone whole brain radiotherapy within 30 days before T-DXd administration and excluding LMC were defined as “analytical active BM”.

### Analytical stable BM by ICR

The patients who were not classified as active and/or LMC by ICR were defined as “stable by ICR”. In this study, those who were defined as stable by ICR and radiated active BM were defined as “analytical stable BM”.

### LMC by ICR

Patients judged to have LMC by ICR were classified as “active with LMC” or “only LMC” and were defined as “LMC” in the analysis.

### Statistical methods

The analysis populations in the present study included the total population, defined as the population who met all the inclusion criteria and did not meet the exclusion criteria (if a patient declined study enrollment before data lock, the patient was excluded from the analysis population), and the population with imaging data of the brain lesion, defined as the population who had multiple imaging data of the brain lesion which were evaluable for IC response assessed by ICR among the total population.

Regarding background patient characteristics, frequencies and percentages were calculated for categorical variables and summary statistics were calculated for continuous data. For OS, TTF, IC-DOR, IC-PFS, and time-to-deterioration of CNS metastasis-related symptoms, the median duration and its 95% CIs were calculated using the Kaplan–Meier method. The point estimates of survival (6 and 12 months) and their 95% CIs were calculated. For ORR, IC-ORR, and IC-CBR, point estimates and their 95% CIs were calculated. Subgroup analyses were performed according to the presence or absence of concomitant BM symptoms and patients’ background characteristics, among others. All statistical analyses were performed using SAS software, version 9.4 (SAS Institute Inc., Cary, NC, USA).

### Supplementary information

Supplementary Materials

nr-reporting-summary

## Data Availability

The datasets used in the current analysis are available from the corresponding author upon reasonable request.
