# Supplementary material for: Treatment with trastuzumab deruxtecan in patients with HER2-positive breast cancer and brain metastases and/or leptomeningeal disease (ROSET-BM)
Source: NPJ Breast Cancer. 2023 Oct 11;9:82. doi: 10.1038/s41523-023-00584-5 (PMC10567705; doi:10.1038/s41523-023-00584-5)
Supplement: Supplementary file 1 — Supplementary Materials [file 41523_2023_584_MOESM1_ESM.docx]

# Supplementary Materials

# Treatment with trastuzumab deruxtecan in patients with HER2-positive breast cancer and brain metastases and/or leptomeningeal disease (ROSET-BM)

Naoki Niikura, et al.

**Supplementary Table 1. Reasons for treatment discontinuation (total population)**

|  | **No. (%)**  ***N*=104** |
| --- | --- |
| Event | 63 (60.6) |
| Discontinuation of trastuzumab deruxtecan treatment | 57 (54.8) |
| Progressive disease | 26 (25.0) |
| Adverse events | 23 (22.1) |
| Interstitial lung disease/lung disorder | 19 (18.3) |
| Adverse events excluding interstitial lung disease^1^ | 4 (3.8) |
| Other | 8 (7.7) |
| Death due to any reason | 1 (1.0) |
| Unknown | 5 (4.8) |

^1^Ulcerative keratitis, malaise, pneumonia, and platelet count decreased in 1 case each.

MedDRA Version 24.0.

**Supplementary Table 2. ORR Based on investigator assessment - systemic including intracranial tumor by RECIST (total population)**

|  |  | **CR**  **No. (%)** | **PR**  **No. (%)** | **SD**  **No. (%)** | **PD**  **No. (%)** |  | **ORR**  **% (95% CI)** |
| --- | --- | --- | --- | --- | --- | --- | --- |
| Total (*n=*97)^1^ | | 5 (5.2) | 49 (50.5) | 32 (33.0) | 11 (11.3) |  | 55.7  (45.2–65.8) |
| Analytical classification of BM^2^ | | | | | | | |
|  | Analytical active BM (*n=*61) | 2 (3.3) | 28 (45.9) | 25 (41.0) | 6 (9.8) |  | 49.2  (36.1–62.3) |
|  | Analytical stable BM (*n=*11) | 1 (9.1) | 7 (63.6) | 2 (18.2) | 1 (9.1) |  | 72.7  (39.0–94.0) |
|  | LMC (*n=*19) | 0 (0.0) | 10 (52.6) | 5 (26.3) | 4 (21.1) |  | 52.6  (28.9–75.6) |
| Clinical presentation of BM | | | | | | | |
|  | Symptomatic (*n=*29) | 2 (6.9) | 13 (44.8) | 10 (34.5) | 4 (13.8) |  | 51.7  (32.5–70.6) |
|  | Asymptomatic (*n=*68) | 3 (4.4) | 36 (52.9) | 22 (32.4) | 7 (10.3) |  | 57.4  (44.8–69.3) |

^1^Of the 104 patients, 7 patients were not evaluable.

^2^Of the 97 patients, 6 patients were not classified by ICR.

*BM* brain metastasis, *CI* confidence interval, *CR* complete response, *LMC* leptomeningeal carcinomatosis, *ORR* overall response rate, *PD* progressive disease, *PR* partial response, *RECIST* Response Evaluation Criteria in Solid Tumors, *SD* stable disease.

**Supplementary Table 3. Study centers**

| **No** | **Name of the institutions** |
| --- | --- |
| 1 | Aichi Cancer Center |
| 2 | Aomori Prefectural Central Hospital |
| 3 | Asahikawa Medical University |
| 4 | Asahikawa-Kosei General Hospital |
| 5 | Izumi City General Hospital |
| 6 | Oita Prefectural Hospital |
| 7 | Ogaki Municipal Hospital |
| 8 | National Hospital Organization Osaka National Hospital |
| 9 | Osaka International Cancer Institute |
| 10 | Okayama Saiseikai General Hospital |
| 11 | Okayama University Hospital |
| 12 | Kasukabe Medical Center |
| 13 | Kanagawa Cancer Center |
| 14 | Yonago Medical Center |
| 15 | Kawasaki Medical School General Medical Center |
| 16 | The Cancer Institute Hospital of Japanese foundation for cancer research |
| 17 | National Cancer Center Hospital |
| 18 | Kansai Medical University Hospital |
| 19 | Kitano Hospital |
| 20 | Gifu University Hospital |
| 21 | National Hospital Organization Kyushu Cancer Center |
| 22 | Kindai University Nara Hospital |
| 23 | Kindai University Hospital |
| 24 | Kumamoto City Hospital |
| 25 | Kurume General Hospital |
| 26 | National Hospital Organization Kure Medical Center and Chugoku Cancer Center |
| 27 | Gunma University Hospital |
| 28 | Kochi Health Sciences Center |
| 29 | National Center for Global Health and Medicine |
| 30 | Saitama Medical University International Medical Center |
| 31 | Saitama Prefectural Cancer Center |
| 32 | Saitama Red Cross Hospital |
| 33 | Saga University Hospital |
| 34 | Juntendo University Shizuoka Hospital |
| 35 | Showa University Hospital |
| 36 | Sapporo City General Hospital |
| 37 | Hakodate Municipal Hospital |
| 38 | National Hospital Organization Takasaki General Medical Center |
| 39 | Tokai University Hospital |
| 40 | Tokyo Kyosai Hospital |
| 41 | Tokyo Metropolitan Cancer and Infectious Diseases Center Komagome Hospital |
| 42 | Tokoname City Hospital |
| 43 | Toranomon Hospital |
| 44 | Nagasaki University Hospital |
| 45 | Niigata Prefectural Central Hospital |
| 46 | Niigata City General Hospital |
| 47 | Hakujyuji Hospital |
| 48 | Hamanomachi Hospital |
| 49 | Higashiyamato Hospital |
| 50 | Hyogo Medical University Hospital |
| 51 | Hyogo Cancer Center |
| 52 | Hiroshima City North Medical Center Asa Citizens Hospital |
| 53 | JA Hiroshima General Hospital |
| 54 | Fukushima Medical University Hospital |
| 55 | Fujieda Municipal General Hospital |
| 56 | Fujisawa City Hospital |
| 57 | National Hospital Organization Hokkaido Cancer Center |
| 58 | Yao Municipal Hospital |
| 59 | Yamagata Prefectural Central Hospital |
| 60 | University of the Ryukyus Hospital |
| 61 | Rinku General Medical Center |
| 62 | National Hospital Organization Nagoya Medical Center |

**Supplementary Table 4. Definitions of outcomes**

| **Best overall response analysis** | | |
| --- | --- | --- |
| **Term** | **Definition** | **Evaluator** |
| ORR | For patients who meet the total population criteria, ORR is the proportion of patients whose best overall response is CR or PR assessed by the ICR and investigators according to RECIST ver.1.1. ORR assessed by the investigators is prioritized. | Investigator |
| IC-ORR | For patients who meet the population with imaging data of the brain lesion criteria, the brain lesion is assessed by ICR according to RECIST ver.1.1. IC-ORR is the proportion of patients whose best overall response is CR or PR. Tumor marker is not considered for CR assessment. | ICR |
| IC-CBR | For patients who meet the population with imaging data of the brain lesion criteria, IC-CBR is the proportion of patients whose best overall response is SD for more than 4 months or 6 months or non-CR or non-PD for more than 4 months or 6 months and more than PR assessed by ICR according to RECIST ver.1.1. | ICR |
| **Survival analysis** | | |
| **Term** | **Definition** | **Evaluator** |
| TTF | For patients who meet the total population criteria, TTF is the duration from the first T-DXd administration to the earliest event for the PD assessment, the treatment discontinuation, or death from all causes. | Investigator |
| PFS | For patients who meet the total population criteria, PFS is the duration from the first T-DXd administration to the earliest event for the PD assessment or death from all causes. | Investigator |
| OS | For patients who meet the total population criteria, OS is the duration from the first T-DXd administration to death from all causes. | Investigator |
| Time-to-deterioration of CNS metastasis-related symptoms | For patients who meet the total population criteria, time-to-deterioration of CNS metastasis-related symptoms is the duration from the first T-DXd administration to the earliest event for the additional usage or increase in steroids or antiepileptic drugs, or first topical treatment for BM. | Investigator |

*BM* brain metastasis, *CBR* clinical benefit rate, *CNS* central nervous system, *CR* complete response, *DOR* duration of response, *IC* intracranial, *ICR* Independent Central Review, *ORR* overall response rate, *OS* overall survival, *PD* progressive disease, *PFS* progression-free survival, *PR* partial response, *RECIST* Response Evaluation Criteria in Solid Tumours, *SD* stable disease, *T-DXd* trastuzumab deruxtecan, *TTF* time-to-treatment failure.

**Supplementary Table 5. Criteria for ICR of brain imaging data^1,2^**

| ***Criteria for active metastasis*** |
| --- |
| The following cases were considered active metastasis: if localized enhancing effects are observed, but cerebral infarction, venous malformation, cavernous hemangioma, and radiation necrosis, among others, are ruled out; if a full or nodular enhancing effect is observed; if the lesion is of a certain size, and cerebral edema may be present around the enhancing lesion; and if a ring-shaped enhancing effect is observed, but stereotactic irradiation was not performed in the same area. In patients with new lesions, active metastasis was suspected when enhanced lesions in the brain, other than vessels, were observed, and active metastasis was determined by subjective evaluations with interpreters. |
| ***Criteria for meningeal carcinomatosis*** |
| Enhancing lesions consistent with brain surface and sulci on contrast-enhanced magnetic resonance imaging (MRI), confirmed by axial and coronal images in two directions whenever possible, and enhancing lesions consistent with cranial nerves on contrast-enhanced MRI were evaluated as meningeal carcinomatosis. |

**Online-only references**

1. Smirniotopoulos, J. G., Murphy, F. M., Rushing, E. J., Rushing, E. J. & Schroeder, J. W. Patterns of contrast enhancement in the brain and meninges. *Radiographics* **27**, 525-551 (2007).

2. Shah, R. et al. Radiation necrosis in the brain: Imaging features and differentiation from tumor recurrence. *Radiographics* **32**, 1343-1359 (2012).

**Supplementary Figures**

**Supplementary Figure 1. Time-to-deterioration of CNS metastasis-related symptoms (total population)**

**
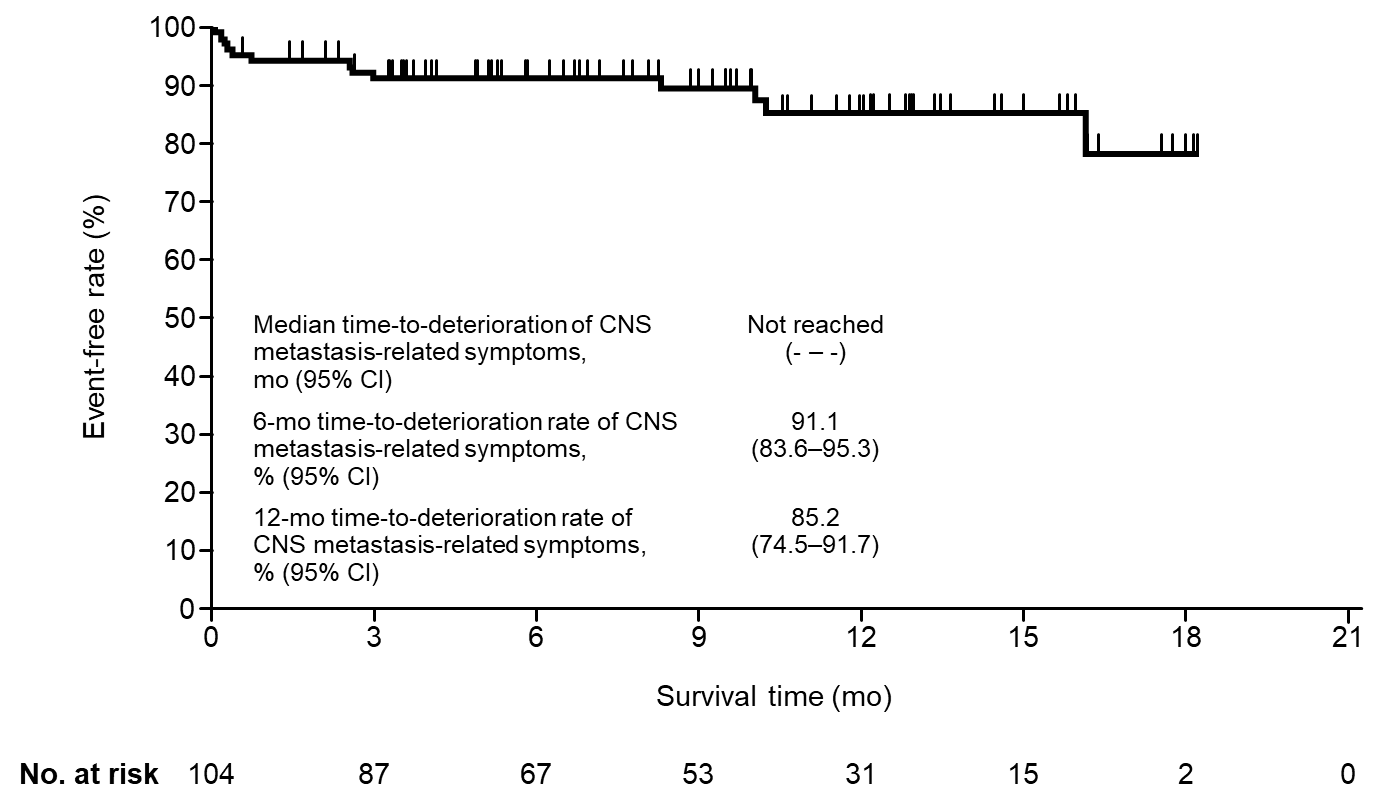
**

*CI* confidence interval, *CNS* central nervous system, *mo* months.

**Supplementary Figure 2. PFS and OS by graded prognostic assessment status (total population)**

**a** PFS, **b** OS. Breast cancer-specific Graded Prognostic Assessment status was categorized into the following subgroups: 1.5–2.5, 3.0, and 3.5–4.0 based on Sperduto PW et al. (Sperduto, P. W. et al. Summary report on the graded prognostic assessment: an accurate and facile diagnosis-specific tool to estimate survival for patients with brain metastases. *J. Clin. Oncol.* **30**, 419-425 (2012)).

*CI* confidence interval, *mo* months, *OS* overall survival, *PFS* progression-free survival.

**Supplementary Figure 3.** **IC-PFS in the brain imaging population and by analytical classification of BM**


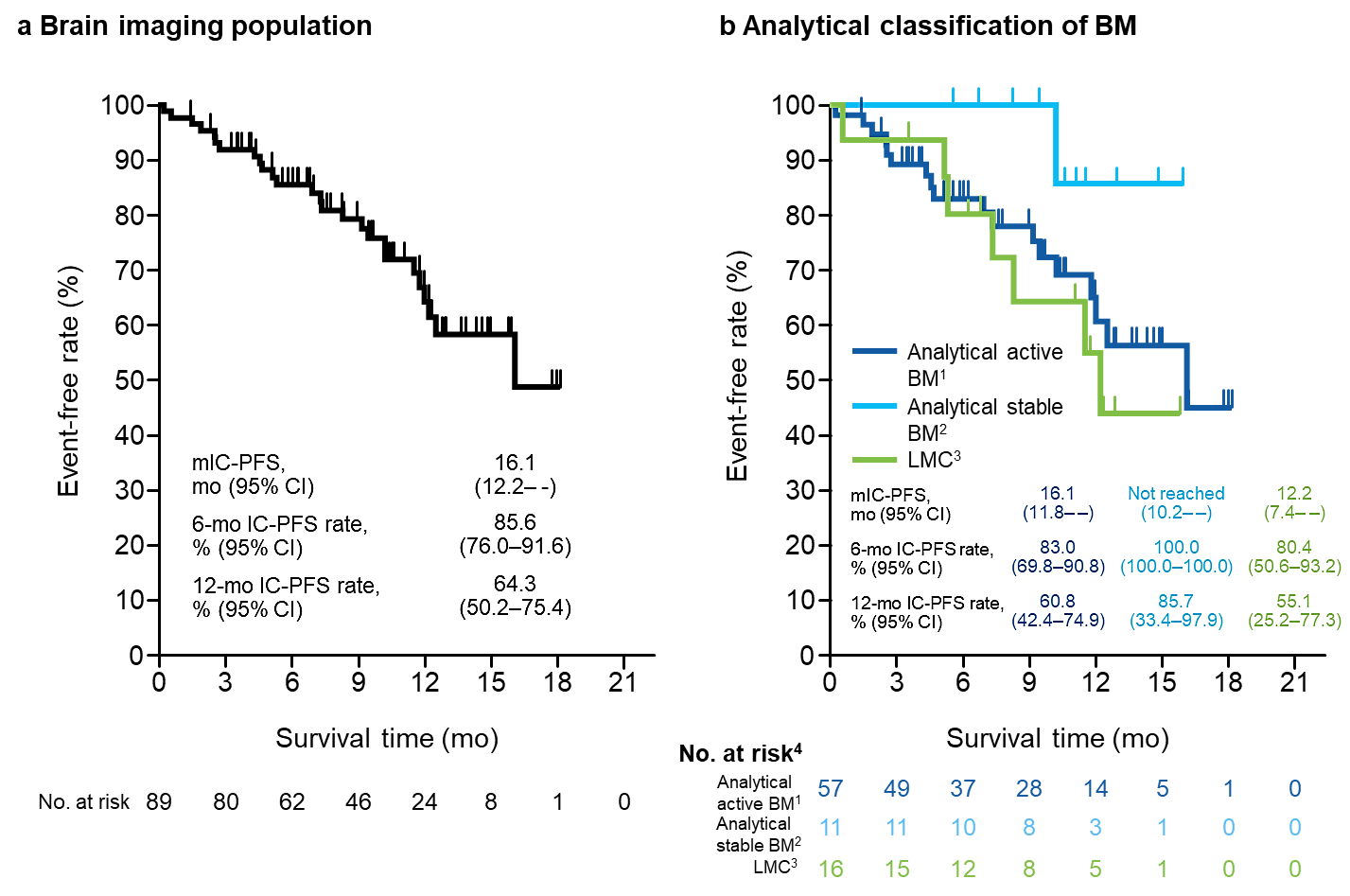


**a** brain imaging population, **b** analytical classification of BM.

^1^Active (not including whole brain radiotherapy within 30 days).

^2^Stable + Active with whole brain radiotherapy within 30 days.

^3^Active with LMC/LMC only.

^4^Of the 89 patients, 5 patients with brain imaging data at baseline were not classified by Independent Central Review.

*BM* brain metastasis, *CI* confidence interval, *IC* intracranial, *LMC* leptomeningeal carcinomatosis, *mo* months, *OS* overall survival, *PFS* progression-free survival.
