# Supplementary material for: Treatment with trastuzumab deruxtecan in patients with HER2-positive breast cancer and brain metastases and/or leptomeningeal disease (ROSET-BM)
Source: NPJ Breast Cancer. 2023 Oct 11;9:82. doi: 10.1038/s41523-023-00584-5 (PMC10567705; doi:10.1038/s41523-023-00584-5)
Supplement: Supplementary file 2 — nr-reporting-summary [file 41523_2023_584_MOESM2_ESM.pdf]

nature portfolio

Corresponding author(s): Naoki Niikura

Last updated by author(s): Jun 5, 2023

Reporting Summary

Nature Portfolio wishes to improve the reproducibility of the work that we publish. This form provides structure for consistency and transparency in reporting. For further information on Nature Portfolio policies, see our Editorial Policies and the Editorial Policy Checklist.

Statistics

For all statistical analyses, confirm that the following items are present in the figure legend, table legend, main text, or Methods section.

n/a	Confirmed	
☐	☒	The exact sample size (*n*) for each experimental group/condition, given as a discrete number and unit of measurement
☐	☒	A statement on whether measurements were taken from distinct samples or whether the same sample was measured repeatedly
☐	☒	The statistical test(s) used AND whether they are one- or two-sided<br>*Only common tests should be described solely by name; describe more complex techniques in the Methods section.*
☒	☐	A description of all covariates tested
☒	☐	A description of any assumptions or corrections, such as tests of normality and adjustment for multiple comparisons
☐	☒	A full description of the statistical parameters including central tendency (e.g. means) or other basic estimates (e.g. regression coefficient) AND variation (e.g. standard deviation) or associated estimates of uncertainty (e.g. confidence intervals)
☒	☐	For null hypothesis testing, the test statistic (e.g. *F*, *t*, *r*) with confidence intervals, effect sizes, degrees of freedom and *P* value noted<br>*Give P values as exact values whenever suitable.*
☒	☐	For Bayesian analysis, information on the choice of priors and Markov chain Monte Carlo settings
☒	☐	For hierarchical and complex designs, identification of the appropriate level for tests and full reporting of outcomes
☒	☐	Estimates of effect sizes (e.g. Cohen's *d*, Pearson's *r*), indicating how they were calculated

Our web collection on statistics for biologists contains articles on many of the points above.

Software and code

Policy information about availability of computer code

Data collection	N/A
Data analysis	N/A

For manuscripts utilizing custom algorithms or software that are central to the research but not yet described in published literature, software must be made available to editors and reviewers. We strongly encourage code deposition in a community repository (e.g. GitHub). See the Nature Portfolio guidelines for submitting code & software for further information.

Data

Policy information about availability of data

All manuscripts must include a data availability statement. This statement should provide the following information, where applicable:

- Accession codes, unique identifiers, or web links for publicly available datasets
- A description of any restrictions on data availability
- For clinical datasets or third party data, please ensure that the statement adheres to our policy

The datasets used in the current analysis are available from the corresponding author upon reasonable request.

Research involving human participants, their data, or biological material

Policy information about studies with human participants or human data. See also policy information about sex, gender (identity/presentation), and sexual orientation and race, ethnicity and racism.

Reporting on sex and gender	This study involved breast cancer patients and the study population primarily consisted of female patients. Therefore, it was not possible to conduct sex-based analysis.
Reporting on race, ethnicity, or other socially relevant groupings	This study was conducted in Japanese patients only.
Population characteristics	See above.
Recruitment	Not applicable as this was a retrospective, medical chart review study.
Ethics oversight	The protocol was approved by the centralized authority (Ethics Review Committee at Tokeikai Kitamachi Clinic), as well as the individual ethics committees at each study center.

Note that full information on the approval of the study protocol must also be provided in the manuscript.

Field-specific reporting

Please select the one below that is the best fit for your research. If you are not sure, read the appropriate sections before making your selection.

☒ Life sciences ☐ Behavioural & social sciences ☐ Ecological, evolutionary & environmental sciences

For a reference copy of the document with all sections, see nature.com/documents/nr-reporting-summary-flat.pdf

Life sciences study design

All studies must disclose on these points even when the disclosure is negative.

Sample size	This was a multicenter, retrospective, medical chart review study. Data from HER2-positive breast cancer patients with BM who received T-DXd treatment between May 25, 2020, and April 30, 2021, were collected from each participating institution using a medical record retrieval system.
Data exclusions	Patients who expressed a desire not to participate in the study prior to data fixation and those who had received T-DXd from participation in a clinical trial were excluded.
Replication	N/A
Randomization	N/A
Blinding	N/A

Reporting for specific materials, systems and methods

We require information from authors about some types of materials, experimental systems and methods used in many studies. Here, indicate whether each material, system or method listed is relevant to your study. If you are not sure if a list item applies to your research, read the appropriate section before selecting a response.

Materials & experimental systems

n/a	Involved in the study	
☒	☐	Antibodies
☒	☐	Eukaryotic cell lines
☒	☐	Palaeontology and archaeology
☒	☐	Animals and other organisms
☐	☒	Clinical data
☒	☐	Dual use research of concern
☒	☐	Plants

Methods

n/a	Involved in the study	
☒	☐	ChIP-seq
☒	☐	Flow cytometry
☒	☐	MRI-based neuroimaging

Clinical data

Policy information about clinical studies

All manuscripts should comply with the ICMJE guidelines for publication of clinical research and a completed CONSORT checklist must be included with all submissions.

Clinical trial registration	UMIN-CTR Clinical Trials, the identifier number UMIN000044995
Study protocol	Not available but can be requested from the corresponding author.
Data collection	Data from HER2-positive breast cancer patients with BM who received T-DXd treatment between May 25, 2020, and April 30, 2021, were collected from 62 medical institutions using a medical record retrieval system. The data cut-off date for survival and other information was October 31, 2021; data entry began on November 1, 2021, and information from medical records was entered retrospectively.
Outcomes	The following outcomes were evaluated for the total population: TTF, PFS, ORR based on investigator assessment, OS, and time-to-deterioration of CNS metastasis-related symptoms. The following outcomes were evaluated for the population with imaging data of the brain lesion: IC-ORR, IC-PFS, IC-DOR, and IC-CBR.
